# Effect of the Multi‐Strain Probiotic SYN‐53 in the Management of Allergic Rhinoconjunctivitis

**DOI:** 10.1111/all.16634

**Published:** 2025-06-27

**Authors:** Karl‐Christian Bergmann, Torsten Zuberbier

**Affiliations:** ^1^ Institute of Allergology Charité Universitätsmedizin Berlin Germany; ^2^ Corporate Member of Freie Universität Berlin Humboldt Universität zu Berlin Berlin Germany; ^3^ Fraunhofer Institute for Translational Medicine and Pharmacology ITMP Immunology and Allergology Berlin Germany

**Keywords:** allergen exposure chamber, allergic rhinitis, allergic rhinoconjunctivitis, microbiome, probiotic

## Abstract

Dysbiosis is increasingly linked to allergy development. This study evaluates the efficacy of the multi‐strain probiotic SYN‐53 in the management of allergic rhinoconjunctivitis (ARC). Eighty‐four subjects with confirmed grass pollen allergy underwent up to three bi‐weekly 3‐day intake cycles with SYN‐53 or placebo. After each cycle, subjects were exposed to grass pollen in an allergen exposure chamber. ARC symptoms were assessed using the Total Symptom Score (TSS) before and after each use of SYN‐53. After one intake cycle, SYN‐53 already showed a trend towards greater efficacy over placebo, which became significant after two cycles (ΔTSS_MAX_: −3.44 ± 0.42 vs. −1.87 ± 0.37; *p* = 0.0067), with 38% vs. 24% symptom relief. In subjects with moderate to severe symptoms, SYN‐53 was already significantly superior after one single intake cycle and improved further after two cycles (ΔTSS_MAX_: −4.78 ± 0.51 vs. −2.43 ± 0.47; *p* = 0.0014), with 45% vs. 26% symptom relief. SYN‐53 is effective in the management of ARC, highlighting the role of bacterial diversity and dosage in probiotic nutritional supplements.

## Graphical abstract

Allergic rhinoconjunctivitis has previously been linked to gut dysbiosis. A double‐blind, placebo‐controlled, randomized clinical trial with the multi‐strain probiotic food supplement SYN‐53 was conducted. Eighty‐four subjects affected by allergic rhinoconjunctivitis were exposed to grass pollen in an allergen exposure chamber to assess safety and efficacy of SYN‐53. SYN‐53 demonstrated significant superiority to placebo in the management of allergic rhinoconjunctivitis. AEC, allergen exposure chamber; ARC, allergic rhinoconjunctivitis; CFU, colony forming units; TSS_MAX_, maximal total symptom score.

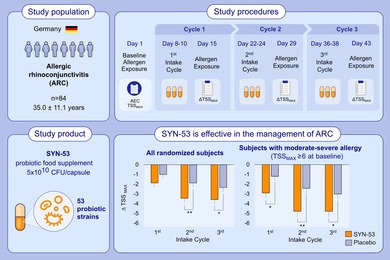

## 1 Introduction

Allergic rhinitis (AR) is a common allergic disorder with a worldwide increasing prevalence [1, 2]. Caused by immunoglobulin E (IgE)‐mediated reactions to inhaled allergens (e.g., plant pollen), AR is characterized by allergic symptoms such as rhinorrhea, nasal itching, sneezing, and nasal congestion. Allergic rhinoconjunctivitis (ARC) refers to the simultaneous appearance of AR and conjunctival inflammation caused by allergic reactions, with itching, burning, redness, and teary eyes [3]. ARC results in marked health and social impairment in affected patients and causes a notable economic burden for health care systems and societies [4]. According to the Global Allergy and Asthma European Network (GA^2^LEN), inadequate allergy treatment leads to annual costs in the range of 55–151 billion euros in the EU alone [5].

Conventional treatment options for ARC include allergen avoidance and pharmacological symptomatic treatment, as well as curative approaches (specific immunotherapy, SIT) [6]. However, they all come with their own limitations: allergen avoidance is impractical, especially when patients are sensitized to multiple allergens [6]. Pharmacological approaches (antihistamines, glucocorticoids) are limited by common side effects including daytime somnolence, sedation, nosebleeding, mouth dryness, drowsiness, and fatigue [7, 8]. Moreover, the effects are not sustained once treatment is discontinued [6]. Allergen immunotherapy is rather arduous and may have substantial side effects, which is one of the reasons for the high rate of patient noncompliance and discontinuation of therapy [9, 10]. Moreover, the effects subside over time. Consequently, there is a high unmet medical need for new, effective, well‐tolerated approaches to manage ARC.

In recent years, research efforts have identified and characterized the important connection between the pathogenesis of allergic diseases and dysbiosis, often characterized by reduced bacterial diversity and/or an altered abundance of certain microbes [11, 12, 13, 14, 15, 16]. In fact, by evaluating fecal 16S rRNA data of 1879 subjects with self‐reported allergies collected by the American Gut Project, Hua and coworkers observed distinct differences in the composition of the gut microbiome (e.g., lower diversity, reduced Clostridiales ssp., and increased Bacteroidales) in subjects with allergies compared to those without, especially in seasonal allergies [17]. Nutritional supplementation with specific beneficial bacteria to restore the natural balance of the gut microbiome thus appears a promising approach to manage allergies. Indeed, animal and in‐human studies have shown that improving the microbial composition through fecal microbiota transplantation (FMT), live biotherapeutic products (LBPs) or probiotics can modulate allergic immune responses and consequently reduce allergic symptoms [15, 18, 19, 20, 21]. Recent meta‐analyses indicate significant improvements in the management of ARC via probiotics, which, however, come along with a high heterogeneity in outcomes. In these studies, probiotic supplementation mainly relies on few (1–3) specific bacterial strains, with multi‐strain probiotics markedly superior to single‐strain approaches [21, 22, 23, 24, 25]. However, the number of strains administered via these multi‐strain probiotics was still markedly below that of FMT, and dosage and administration regime differed considerably from FMT. Although FMT of healthy donors may deliver a multitude of beneficial bacterial strains, it can pose significant safety risks due to the potential transmission of pathogenic organisms or undetected infectious agents. Further, its application remains impractical due to challenges in standardizing donor selection, ensuring consistent composition, and optimizing delivery methods impeding the applicability of this method on a large scale [26, 27].

Building on this, SYN‐53 was designed as a nutritional supplement containing 53 distinct viable bacterial strains, delivered at a daily dose of 1.5 × 10^11^ colony forming units (CFU). The administered bacterial strains were carefully selected as natural inhabitants of a healthy gastrointestinal tract. The aim of this double‐blind, placebo‐controlled clinical trial was to determine the efficacy of the multi‐strain probiotic SYN‐53 in the management of ARC. In this study, a state‐of‐the‐art allergen exposure chamber (AEC) was used to perform allergen provocation testing to assess ARC symptoms.

## 2 Methods

### 2.1 Study Design

The study was performed as a double‐blind, placebo‐controlled, monocentric, randomized, parallel‐group clinical study. It took place in the GA^2^LEN AEC at the European Centre for Allergy Research Foundation (ECARF) Institute (Berlin, Germany) during the months of October through December 2020 to avoid the confounding influence of the pollen season.

The study was divided into a screening phase (*V*_0_) to recruit potential subjects with ARC, followed by a baseline exposure on visit 1 (exposure 1; *E*_1_) in the AEC to determine the ITT (intention‐to‐treat) population.

**FIGURE 1:**
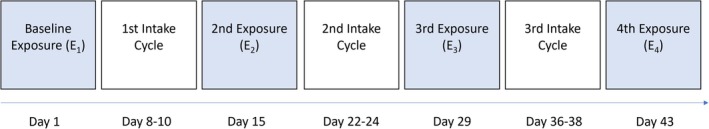
Timeline of the intake cycles and allergen exposures during the clinical study.

All eligible study subjects underwent up to three bi‐weekly 3‐day intake cycles with SYN‐53 or placebo. Each cycle was followed by an exposure in the AEC 5 days post intake. A 2‐week interval separated each cycle. Safety phone calls were conducted 1 day after each allergen exposure (not shown). The process of repeated intake cycles and allergen exposures is depicted in Figure 1.

To explore the required number of intake cycles to reach a significant symptom relief, statistical analysis was conducted after each exposure in the AEC. All statistical analyses were performed by a statistician independent of study conduct to maintain double blindness.

For all AEC‐exposures, standardized grass pollen ( *Phleum pratense* , Allergon AB, Ängelholm, Sweden) was used. At each exposure, subjects were exposed to 4.000 grass pollen/m^3^ for 120 min at room temperature (20°C) and 55% relative humidity.

The study protocol was approved by the ethics committee of the Ethics Commission of Charité Universitätsmedizin, Berlin, (EA1/216/20) and was conducted in accordance with the Declaration of Helsinki. The trial was registered with the ISRCTN registry (ISRCTN99056955). All subjects provided informed consent.

### 2.2 Participants

The study population consisted of individuals with clinically relevant grass pollen sensitization. Individuals aged between 18 and 65 years with a known allergy history of more than 2 years and a positive skin prick test (wheal diameter ≥ 3 mm) were admitted to baseline exposure (*E*_1_) in the AEC and randomized if their maximum Total Symptom Score TSS_MAX_ was ≥ 3 (cf. Section 2.3 for a definition of the TSS).

Exclusion criteria included acute infections, current cancer diagnosis, autoimmune disease, gastrointestinal diseases causing diminished metabolic processing of orally taken substances, or severe types of the following chronic diseases: neurologic diseases, metabolic diseases, severe asthma, severe pulmonary obstruction, innate anomalies of the heart, gastrointestinal tract, or lung. Furthermore, people suffering from psychological diseases (e.g., depression), eating disorders (e.g., bulimia), alcohol or drug addiction, allergic reactions to any of the ingredients of the formulations tested, and contraindications against adrenaline or any other rescue medication (especially cetirizine) were excluded. In addition, pregnant or breastfeeding women, heavy smokers (more than 20 cigarettes/day), people who had undergone specific immune therapy in the past 5 years, and people who had participated in clinical trials in the past 3 months prior to the screening procedure were not admitted to the study.

The following medications were not permitted throughout the study (applicable elimination time prior to the study): decongestant nasal drops (3 days), antihistamines (5 days), anti‐allergic eye drops and nasal sprays (1 week), topical steroids (2 weeks), systemic corticosteroids (3 weeks), probiotics (4 weeks), antibiotics (4 weeks).

### 2.3 Outcome Parameters

The primary outcome measure was the well‐established maximum “Total Symptom Score” (TSS_MAX_) described in Pfaar et al. [28] TSS is composed of the Total Eye Symptom Score (TESS, max. 12 points) and the Total Nasal Symptom Score (TNSS, max. 12 points) with a maximum possible score of 24 points (cf. Table 1). The TNSS consisted of itching, sneezing, secretion, and nasal obstruction. Symptoms of the TESS were itching, foreign body feeling, tears, and eye redness.

**TABLE 1:** Detailed description of symptoms covered by the Total Symptom Score (TSS).

Total Symptom Score (TSS)—max. 24 points	
Total Nasal Symptom Score (TNSS)—max. 12 points	Total Eye Symptom Score (TESS)—max. 12 points
Itching—max. 3 points	Itching—max. 3 points
Sneezing—max. 3 points	Foreign body feeling—max. 3 points
Secretion—max. 3 points	Teary eyes—max. 3 points
Nasal obstruction—max. 3 points	Redness—max. 3 points

TSS was serially measured every 10 min during the 120‐min exposure. The intensity of each individual symptom was evaluated on a scale ranging from 0 to 3 (0 = no symptoms; 1 = mild symptoms; 2 = moderate symptoms; 3 = severe symptoms), summing up to a maximum of 12 points each for TESS and TNSS, respectively (cf. Table 1). TSS_MAX_ is defined as the highest TSS value measured during the 120‐min exposure.

Additional parameters included further symptom‐related metrics such as the Total Other Symptom Score (TOSS) and the Total Bronchial Symptom Score (TBSS), each measured every 10 min, the visual analogue scale (VAS) to measure overall well‐being (every 30 min), the Peak Nasal Inspiratory Flow (PNIF), and nasal secretion. The occurrence of adverse events was monitored throughout the study.

### 2.4 Study Product

SYN‐53 is a multi‐strain, multi‐species probiotic nutritional supplement containing 53 viable probiotic strains that are ubiquitous inhabitants of the healthy human gut microbiome. Each capsule contains at least 5 × 10^10^ colony forming units (CFU). In addition, the supplement contains vitamin B12, biotin, maize starch (filling agent), hydroxypropylmethyl cellulose (capsule shell), and the anti‐caking agents magnesium stearate and silicon dioxide. One intake cycle consisted of three capsules per day, taken at mealtimes for three consecutive days.

The placebo consisted of inulin and maize starch (filling agents), hydroxypropylmethyl cellulose (capsule shell), and the anti‐caking agents magnesium stearate and silicon dioxide. SYN‐53 and the placebo were indistinguishably packaged and differed neither in appearance nor taste.

The emergency medication was one tablet of cetirizine (10 mg), which could be taken within 24 h after an AEC session if required.

### 2.5 Randomization and Masking

Participants were randomly assigned to either the SYN‐53 or the placebo arm following a block randomization list generated by an unmasked randomization administrator who was independent of study conduct and data analysis. Participants, investigators, clinical monitors, project managers, and statisticians were all masked to the randomization. Sealed emergency envelopes that contained each participant's study group allocation were stored safely at the study site for emergency unblinding if necessary.

### 2.6 Statistical Analysis

To determine the effectiveness of SYN‐53, the change of the TSS_MAX_ (∆TSS_MAX_) during exposure after each intake cycle compared to TSS_MAX_ recorded at baseline (*E*_1_) was determined. Change in TSS_MAX_ per participant from baseline exposure (*E*_1_) to exposure after the following intake cycles was calculated as follows:

$${\text{∆TSS}}_{\mathrm{MAX}}\;\left(E_{k},E_{1}\right)={\mathrm{TSS}}_{\mathrm{MAX}}\left(E_{k}\right)-{\mathrm{TSS}}_{\mathrm{MAX}}\left(E_{1}\right)\;\left(k=2,3,4\right)$$

Normality of the data were tested using Shapiro‐Wilk Test with a significance level of *p* < 0.05. Based on the results, normal distribution was assumed. Obtained data were analyzed by covariance analysis (ANCOVA) setting the change from the initial score as the dependent variable, the investigational product as a factor, and baseline score as a covariate.

In order to maintain the overall level of *α* < 0.05 in the multiple test situation, the significance level for a test according to the Bonferroni method (p* < *α*/*n*) was set to *p* < 0.0166.

Missing data points were replaced by single imputation (Last Observation Carried Forward, LOCF). Multiple imputation (MI), Baseline Observation Carried Forward (BOCF) and complete case analysis (CC) yield similar results (cf. Table S1).

A priori, subjects were clustered into symptom severity groups based on TSS_MAX_ at baseline (TSS_MAX_ ≥ *x*, where each *x* is an integer ≥ 3) to investigate whether symptom alleviation depends on symptom severity at baseline. Special focus was laid on patients with at least moderate symptoms (TSS_MAX_ ≥ 6), who would clinically benefit the most.

Additionally, a responder analysis was conducted post hoc. Building on Pfaar et al. [28], responders were defined as subjects with TSS_MAX_ ≥ 6 that (i) showed a TSS_MAX_ improvement of at least 30% against baseline and (ii) whose TSS_MAX_ fell below the threshold (TSS_MAX_ ≤ 6). The responder analysis was evaluated using Fisher's Exact Test.

Tolerability and safety were evaluated based on the frequency and severity of adverse events, as well as on the need for emergency medication and discontinuations of intake. This analysis was performed on the safety population, which contains those subjects that received at least one dose of study medication.

All statistical data analyses were done by an independent biostatistician using SAS software version 9.4. Additional graphics were done using the R package (version 4.1.2 or later).

## 3 Results

A total of 90 subjects with a history of grass pollen allergy and a positive skin prick test were recruited and screened for TSS_MAX_ ≥ 3 at baseline (*E*_1_) in the AEC. Out of these 90 subjects, 84 fulfilled TSS_MAX_ ≥ 3 and were randomized (intention‐to‐treat population, ITT). ITT subjects were on average 34.9 ± 11.2 years old and 63% were female (cf. Table 2A).

**TABLE 2:** Baseline characteristics of the intention‐to‐treat (ITT) population (A) and the study subjects of the cluster TSS_MAX_ ≥ 6 (B).

	SYN‐53	Placebo	Total
(A)			
Gender *N* (%)			
F	23 (62.2)	30 (63.8)	53 (63.1)
M	14 (37.8)	17 (32.2)	31 (36.9)
Age (year)	33.1 ± 11.05	35.7 ± 11.4	34.9 ± 11.2
Height (cm)	173.8 ± 9.2	172.3 ± 10.0	173.0 ± 9.7
Weight (kg)	75.2 ± 16.7	74.0 ± 14.0	74.6 ± 15.1
BMI	24.7 ± 4.2	25.0 ± 4.5	24.9 ± 4.3
(B)			
Gender *N* (%)			
F	18 (64.3)	21 (63.6)	39 (63.9)
M	10 (35.7)	12 (36.4)	22 (36.1)
Age (year)	34.8 ± 11.1	35.2 ± 11.3	35.0 ± 11.1
Height (cm)	172.7 ± 8.6	171.9 ± 10.4	172.2 ± 9.6
Weight (kg)	76.2 ± 17.4	72.5 ± 12.9	74.2 ± 15.1
BMI	25.3 ± 4.4	24.6 ± 4.2	25.0 ± 4.3

*Note:* Data presented as mean ± SD.

A subgroup of 61 subjects had moderate‐to‐severe symptoms (TSS_MAX_ ≥ 6) at baseline. These subjects were on average 35.0 ± 11.1 years old and 64% were female (cf. Table 2B).

Baseline exposure data (*E*_1_) data did not show significant differences between the verum and placebo groups in either the cluster TSS_MAX_ ≥ 3 (TSS_MAX_ [mean ± SEM]: 8.95 ± 4.28 vs. 7.94 ± 3.37; *p* = 0.3400) or the cluster TSS_MAX_ ≥ 6 (TSS_MAX_ [mean ± SEM]: 10.57 ± 3.60 vs. 9.52 ± 2.73; *p* = 0.2859).

### 3.1 Results for the ITT Population (TSS_MAX_ ≥ 3)

**FIGURE 2:**
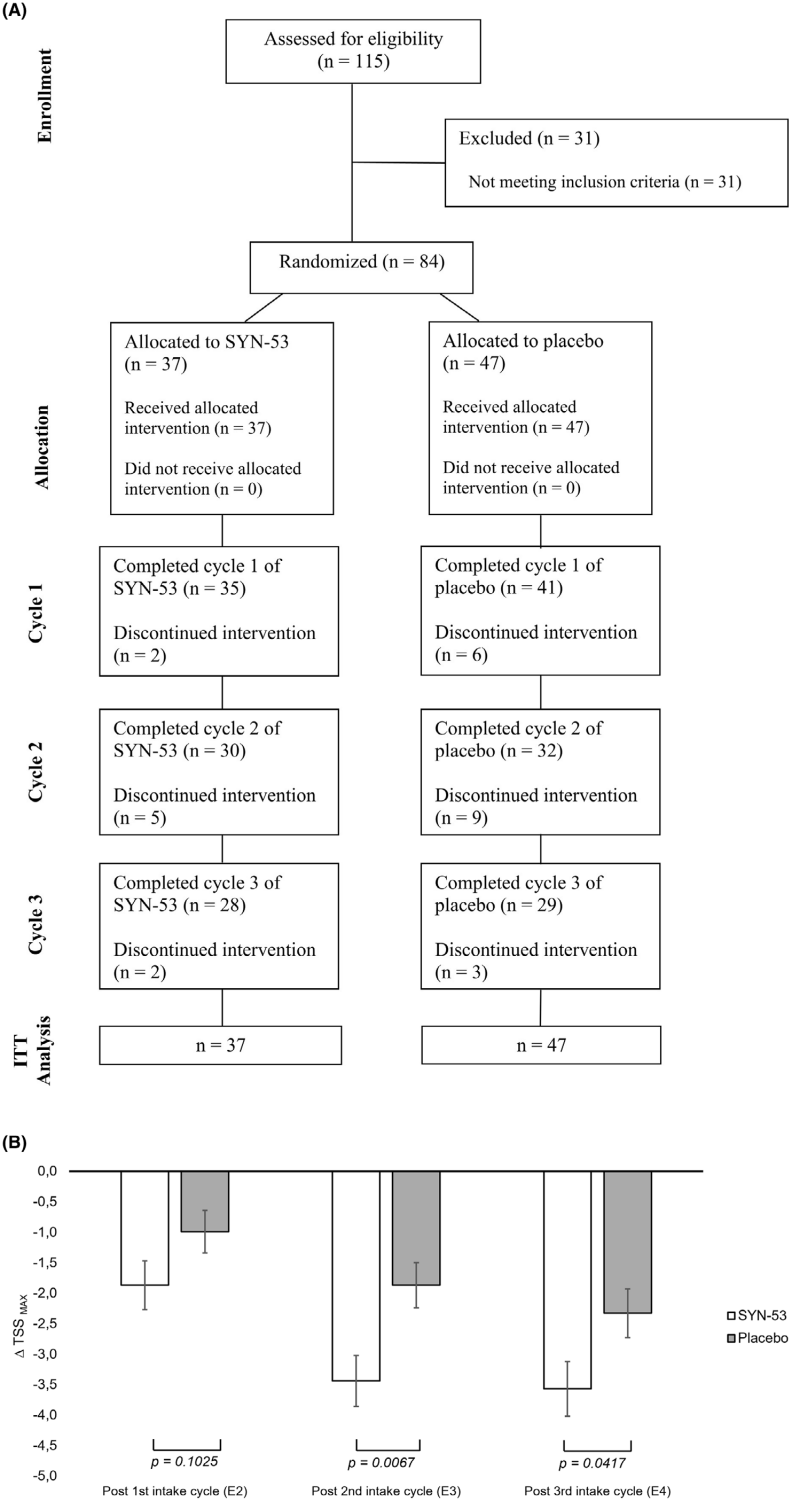
Overview and results of the intention‐to‐treat (ITT) population. (A) Overview of the subject flow (TSS_MAX_ ≥ 3). (B) Difference in maximum Total Symptom Score (∆TSS_MAX_) in the ITT population after one, two and three intake cycles. ANCOVA analysis, values shown as mean ± SEM, *n*(placebo) = 47, *n*(intervention) = 37.

Out of the 84 randomized subjects (ITT population), 37 were randomly assigned to receive SYN‐53 and 47 to receive placebo. 57 subjects completed three further intake cycles: 28 (75.7%) of the SYN‐53 group and 29 (61.7%) of the placebo group with subsequent exposures (*E*_2_, *E*_3_, *E*_4_) in the AEC. During the study, a total of 27 subjects dropped out due to various reasons that were all unrelated to SYN‐53. The main reasons for dropout were related to the COVID‐19 pandemic (affected or contact person), other common illnesses, personal reasons, or without any explanation (cf. Figure 2A). Missing data points due to dropouts were imputed for ITT analysis.

In the ITT population, a trend favoring SYN‐53 was evident after a single cycle (∆TSS_MAX_ [mean ± SEM]: −1.87 ± 0.40 vs. −0.99 ± 0.35; *p* = 0.1025) and reached significance after a second cycle (∆TSS_MAX_ [mean ± SEM]: −3.44 ± 0.42 vs. −1.87 ± 0.37; *p* = 0.0067). Following a third cycle, the effects stabilized further in favor of SYN‐53 (∆TSS_MAX_ [mean ± SEM]: −3.57 ± 0.45 vs. −2.33 ± 0.40; *p* = 0.0417) (cf. Figure 2B) but were less pronounced due to a steadily evolving rise in the placebo effect—a phenomenon also observed in the cluster TSS_MAX_ ≥ 6.

Similarly, responder rates were 32.4% vs. 19.1% in favor of SYN‐53 after the first cycle (*p* = 0.207), 45.9% vs. 29.8% in favor of SYN‐53 after a second cycle (*p* = 0.172) and still in favor of SYN‐53 after a third cycle (48.6% vs. 36.2%, *p* = 0.273).

The analysis after MI and BOCF imputation as well as the CC analysis yielded similar results and significance levels (cf. Table S1).

### 3.2 Results for Cluster TSS_MAX_ ≥ 6

**FIGURE 3:**
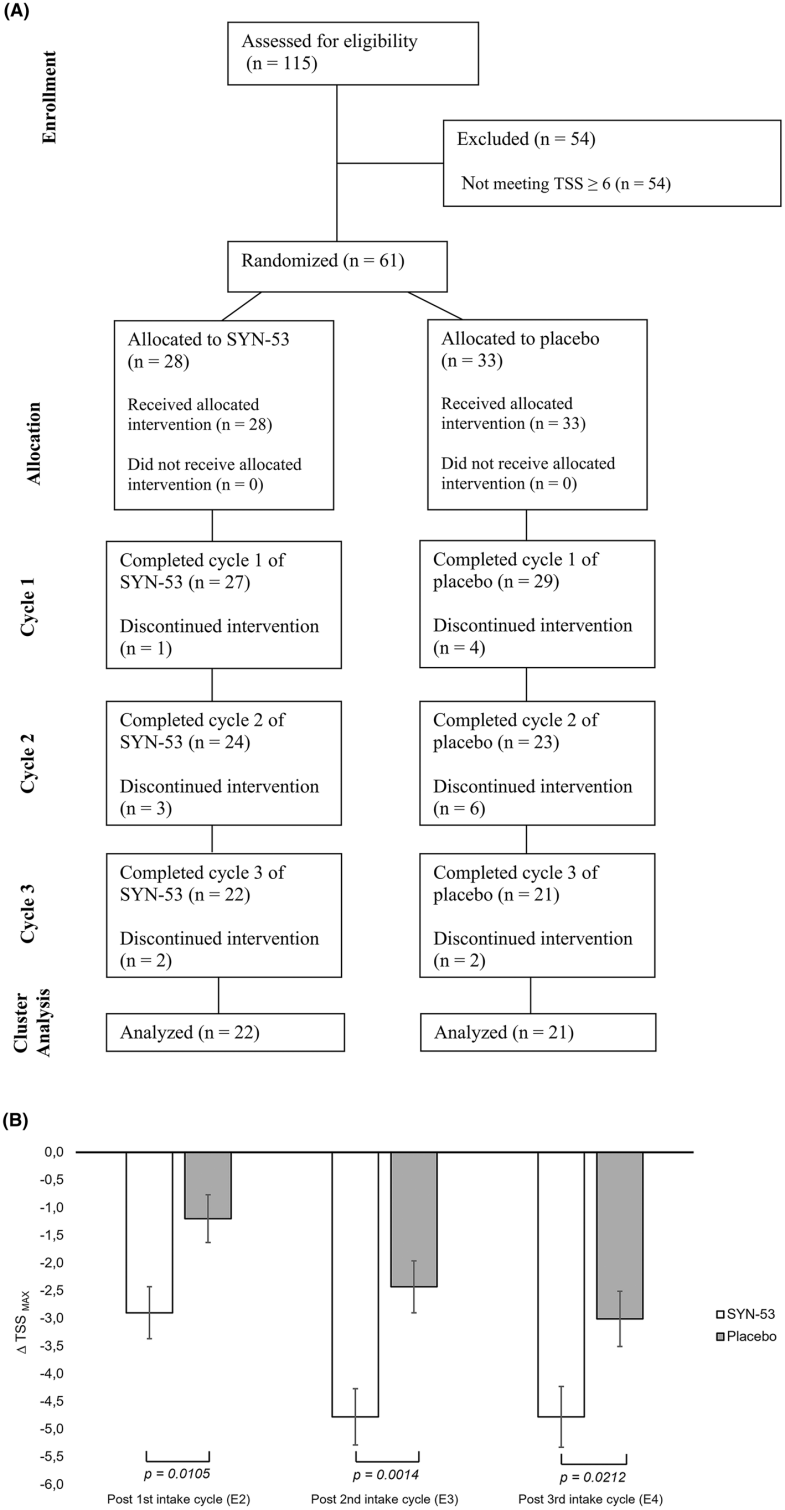
Overview and results of the subgroup analysis, with subject cluster TSS_MAX_ ≥ 6. (A) Overview of the subject flow of cluster TSSMAX ≥ 6. (B) Difference in maximum Total Symptom Score (∆TSS_MAX_) in the cluster TSSMAX ≥ 6 after one, two and three intake cycles. ANCOVA analysis, values shown as mean ± SEM, *n*(placebo) = 33, *n*(intervention) = 28.

28 of the 61 subjects in the cluster TSS_MAX_ ≥ 6 were randomly assigned to receive SYN‐53 and 33 to receive placebo. Of those 61 subjects, 43 (22 (78.6%) of the SYN‐53 group and 21 (63.6%) of the placebo group) completed three further intake cycles with subsequent exposures (*E*_2_, *E*_3_, *E*_4_) in the AEC. During the study, a total of 18 subjects dropped out due to the COVID‐19 pandemic (affected or contact person), other common illnesses, personal reasons, or without explanation (cf. Figure 3A). Missing data were imputed.

In the subgroup of subjects with moderate‐to‐severe symptoms (TSS_MAX_ ≥ 6) at baseline, SYN‐53 was significantly superior to placebo after just one intake cycle (∆TSS_MAX_ [mean ± SEM]: −2.90 ± 0.47 vs. −1.20 ± 0.43; *p* = 0.0105). This effect was further strengthened after a second cycle (∆TSS_MAX_ [mean ± SEM]: −4.78 ± 0.51 vs. −2.43 ± 0.47; *p* = 0.0014). Following a third cycle, effects stabilized in favor of SYN‐53 (∆TSS_MAX_ [mean ± SEM]: −4.78 ± 0.55 vs. −3.01 ± 0.50; *p* = 0.0212) (cf. Figure 3B) but were less pronounced due to a steadily evolving rise in the placebo effect.

Accordingly, after a single intake cycle, responder rates were in favor of SYN‐53 at 39.3%, compared with 15.2% in the placebo group (*p* = 0.0433). These findings were even further pronounced after a second cycle, where responder rates in the SYN‐53 group increased to 60.7% vs. 27.3% in the placebo group (*p* = 0.011). The results after a third cycle were still distinctly in favor of SYN‐53 (50%) vs. placebo (33.3%), yet non‐significant (*p* = 0.205).

In the safety population, a total of 23 adverse events (12 in the SYN‐53 group [52.17%] and 11 in the placebo group [47.8%]) were reported. Of these adverse events, 82.6% were graded as mild and 17.4% as moderate (cf. Table S2) with no relevant differences between SYN‐53 and placebo. None of the events were serious and all emergency envelopes were returned to the sponsor unopened, confirming that masking was maintained throughout the study.

47.8% of the events led to discontinuation of investigational product use, with no significant differences between placebo and SYN‐53.

Most adverse events were gastrointestinal in nature (abdominal pain, diarrhea, nausea), followed by infections and infestations (nasophayngitis). Ten adverse events were regarded as related to investigational product use (5 SYN‐53, 5 placebo) with no difference between both arms (placebo, SYN‐53) (cf. Table 3).

**TABLE 3:** Detailed view on adverse events for the placebo and SYN‐53 study groups.

	Investigational product		
	Placebo, *N*	SYN‐53, *N*	All, *N*
Gastrointestinal disorders			
Abdominal pain	2	2	4
Abdominal pain upper	2	1	3
Diarrhea	1	2	3
Nausea	1		1
All	6	5	11
Infections and infestations			
Nasopharyngitis	3	5	8
All	3	5	8
Injury, poisoning and procedural complications			
Wound	1		1
All	1		1
Musculoskeletal and connective tissue disorders			
Rheumatoid arthritis		1	1
All		1	1
Renal and urinary disorders			
Cystitis noninfective		1	1
All		1	1
Respiratory, thoracic and mediastinal disorders			
Asthma	1		1
All	1		1
All	11	12	23

## 4 Discussion

To the best of our knowledge, SYN‐53 is the first probiotic supplement that demonstrated efficacy in the management of ARC within one single three‐day intake cycle in a double‐blind placebo‐controlled clinical study.

While meta‐analyses of clinical trials on the management of ARC with probiotics suggest that probiotic supplementation seems effective overall, there is high heterogeneity in outcomes and sample sizes are often small [21, 25]. Further, probiotics investigated in the management of ARC consisted of a maximum of three bacterial strains, mostly at concentrations in the range of ~10^7^–10^9^ CFU/day, continuously administered over periods ranging from multiple weeks up to a year.

The approach taken for SYN‐53 in this study stands out for three key reasons:

1. It contains a highly diverse composition of 53 bacterial strains.
2. It delivers a concentration as elevated as 5 × 10^10^ CFU per capsule.
3. It is administered in a sequence of consecutive three‐day intake cycles.

The results of this study reveal a strong connection between probiotic supplement administration regimes, dosage, and diversity and their efficacy in the management of allergies. The fact that one single intake cycle led to a significant symptom reduction deserves particular attention, given the widespread assumption that a continuous, daily use of probiotic preparations for several weeks or months is necessary to achieve a significant symptom amelioration [21]. The high‐dose, multi‐strain preparation and short‐term intake regime of SYN‐53 represents an innovative approach for patients suffering from symptoms of ARC.

SYN‐53 represents an alternative nutritional approach in the management of ARC, which yields results that are comparable to the effects of conventional treatment options, which, however, are often associated with various side effects such as daytime somnolence, sedation, drowsiness, and fatigue [7, 8, 29, 30]. As an advantage to the latter, the nutritional supplement SYN‐53 is well tolerated, and adverse events were not different from placebo. SYN‐53 is hence suitable for long‐term use.

Besides its randomized, double‐blind, placebo‐controlled design, the strength of this study is the controlled nature of the AEC, which improves the reliability and comparability of results. The AEC was developed to counteract typical problems of clinical trials during pollen season, where natural allergen exposure can vary in concentration, duration, and purity [31, 32]. In addition, the study design minimized the influence of allergic symptoms due to seasonal pollen symptoms.

A potential limitation of this study is the considerable placebo effect after the third intake cycle, which, however, is not uncommon in clinical trials in allergies [33]. In addition, we experienced a high drop‐out rate due to the COVID‐19 (SARS‐CoV‐2 virus) pandemic and the obligatory contact restrictions and isolation measures that were in place in Germany at the time the study was conducted.

Overall, the study shows that the novel intake regime, in combination with the formulation of the 53 bacterial strains contained in SYN‐53, is both effective in the management of ARC and well tolerated. The results presented in this study pave the way for further research. Specifically, a detailed analysis of the individual contribution of diversity, dosage, and intake regime in the design of probiotics deserves further investigation.

## Author Contributions

T.Z. and K.C.B. made substantial contributions to the conception and design of the study, and to the acquisition, analysis, and interpretation of data. Both authors were equally involved in drafting the manuscript. They agree to the version to be published and agree to be accountable for all aspects of the work, ensuring that any questions related to the accuracy or integrity of any part of the work are appropriately investigated and resolved. The sponsor participated in the study design. Data collation and statistical analysis were conducted by an independent clinical research organization (CRO).

## Conflicts of Interest

The European Centre for Allergy Research Foundation (ECARF) received reimbursement for participating as a study center. K.‐C.B. received honoraria for lectures from ALK, Allergopharma, Almirall, AstraZeneca, Bencard, Berlin‐Chemie, Chiesi, GSK, HAL, Mundipharma, Novartis, Sanofi, and Stallergenes during the last 5 years. T.Z. has received institutional funding for research and/or honoria for lectures and/or consulting from Amgen, AstraZeneca, AbbVie, ALK, Almirall, Astellas, Bayer Health Care, Bencard, Berlin Chemie, Blueprint Medicines, FAES, HAL, Henkel, Kryolan, Leti, L'Oreal, Meda, Menarini, Merck, MSD, Novartis, Pfizer, Sanofi, Stallergenes, Takeda, Teva and UCB, Uriach; in addition, he is a member of ARIA/WHO, DGAKI, ECARF, GA2LEN and WAO.

## Supporting information


Table S1.
Table S2.

## Data Availability

The data that support the findings of this study are available on request from the corresponding author. The data are not publicly available due to privacy or ethical restrictions.
