# Supplementary material for: Effect of the Multi‐Strain Probiotic SYN‐53 in the Management of Allergic Rhinoconjunctivitis
Source: Allergy. 2025 Jun 27;80(8):2158–66. doi: 10.1111/all.16634 (PMC12368763; doi:10.1111/all.16634)
Supplement: Supplementary file 1 — Table S1. Table S2. [file ALL-80-2158-s001.docx]

# Title

# **Effect of the multi-strain probiotic SYN-53 in the management of allergic rhinoconjunctivitis**

Karl-Christian Bergmann^1,2^and Torsten Zuberbier^1,2*^

1. Institute of Allergology, Charité Universitätsmedizin, Berlin, Corporate Member of Freie Universität Berlin and Humboldt Universität zu Berlin, Berlin, Germany
2. Fraunhofer Institute for Translational Medicine and Pharmacology ITMP, Allergology and Immunology, Berlin, Germany

*Corresponding Author

**Supplementary material**

|  |  | ∆**TSS_MAX_** | | | | | |
| --- | --- | --- | --- | --- | --- | --- | --- |
|  |  | ***N*** | **Exposure after 1^st^ intake cycle (E_2_)** | ***N*** | **Exposure after 2^nd^ intake cycle (E_3_)** | ***N*** | **Exposure after 3^rd^ intake cycle (E_3_)** |
| **BOCF** | **SYN-53** | *37* | -1.87 ± 0.40 | *37* | -3.15 ± 0.45 | *37* | -2.90 ± 0.48 |
|  | **Placebo** | *47* | -0.99 ± 0.35 | *47* | -1.65 ± 0.35 | *47* | -2.21 ± 0.42 |
|  | ***p value*** |  | *0.1025* |  | *0.0146* |  | *0.2895* |
| **MI** | **SYN-53** | *37* | -1.92 ± 0.43 | *37* | -3.64 ± 0.51 | *37* | -3.72 ± 0.44 |
|  | **Placebo** | *47* | -1.08 ± 0.39 | *47* | -2.33 ± 0.49 | *47* | -3.52 ± 0.43 |
|  | ***p value*** |  | *0.1473* |  | *0.0653* |  | *0.7411* |
| **CC** | **SYN-53** | *35* | -2.00 ± 0.42 | *30* | -3.85 ± 0.48 | *28* | -3.79 ± 0.47 |
|  | **Placebo** | *41* | -1.13 ± 0.39 | *32* | -2.46 ± 0.47 | *29* | -3.61 ± 0.46 |
|  | ***p value*** |  | *0.1361* |  | *0.0455* |  | *0.7887* |

*Table S1: ANCOVA results for ∆TSS_MAX_ after one, two and three intake cycles after various data replacement methods. BOCF = baseline observation carried forward, MI = multiple imputation, CC = complete cases. Data is presented as Mean ± SEM.*

| **Severity** | **Placebo N** | **SYN-53 N** | **All N** |
| --- | --- | --- | --- |
| mild | 9 | 10 | 19 |
| moderate | 2 | 2 | 4 |
| **All** | **11** | **12** | **23** |

Table S2: Summary of adverse events.
